# Studying human behavior with virtual reality: The Unity Experiment Framework

**DOI:** 10.3758/s13428-019-01242-0

**Published:** 2019-04-22

**Authors:** Jack Brookes, Matthew Warburton, Mshari Alghadier, Mark Mon-Williams, Faisal Mushtaq

**Affiliations:** 1 School of Psychology, University of Leeds, Leeds, West Yorkshire UK; 2 Centre for Immersive Technologies, University of Leeds, Leeds, West Yorkshire UK

**Keywords:** Virtual reality, Unity, Software, Experiment, Behavior, Toolkit

## Abstract

Virtual reality (VR) systems offer a powerful tool for human behavior research. The ability to create three-dimensional visual scenes and to measure responses to the visual stimuli enables the behavioral researcher to test hypotheses in a manner and scale that were previously unfeasible. For example, a researcher wanting to understand interceptive timing behavior might wish to violate Newtonian mechanics so that objects can move in novel 3-D trajectories. The same researcher might wish to collect such data with hundreds of participants outside the laboratory, and the use of a VR headset makes this a realistic proposition. The difficulty facing the researcher is that sophisticated 3-D graphics engines (e.g., Unity) have been created for game designers rather than behavioral scientists. To overcome this barrier, we have created a set of tools and programming syntaxes that allow logical encoding of the common experimental features required by the behavioral scientist. The Unity Experiment Framework (UXF) allows researchers to readily implement several forms of data collection and provides them with the ability to easily modify independent variables. UXF does not offer any stimulus presentation features, so the full power of the Unity game engine can be exploited. We use a case study experiment, measuring postural sway in response to an oscillating virtual room, to show that UXF can replicate and advance upon behavioral research paradigms. We show that UXF can simplify and speed up the development of VR experiments created in commercial gaming software and facilitate the efficient acquisition of large quantities of behavioral research data.

Virtual reality (VR) systems are opening up new opportunities for behavioral research, because they allow visual (and auditory) stimuli to be displayed in 3-D computer-generated environments that can correspond to the participant’s normal external Cartesian space, but that do not need to adhere to the rules of Newtonian mechanics (Wann & Mon-Williams, 1996). Moreover, VR systems support naturalistic interactions with virtual objects and can provide precise measures of the kinematics of the movements made by adults and children in response to displayed visual stimuli. In addition, the relatively low cost and portability of these systems lowers the barriers to performing research in nonlaboratory settings.

The potential advantages of VR in behavioral research have been recognized for at least two decades (e.g., Loomis, Blascovich, & Beall, 1999), but recent advantages in technology and the availability of hardware and software are making VR a feasible tool for all behavioral researchers (rather than for a limited number of specialist VR labs). For example, researchers can now access powerful software engines that allow the creation of rich 3-D environments. One such popular software engine is Unity (alternatively called Unity3D; Unity Technologies, 2018). Unity is a widely used 3-D game engine for developing video games, animations, and other 3-D applications, and it is growing in ubiquity. It is increasingly being used in research settings as a powerful way of creating 3-D environments for a range of applications (e.g., psychology experiments, surgical simulation, or rehabilitation systems). The recent popularity of VR head-mounted displays (HMDs) has meant that Unity has become widely used by game developers for the purpose of crating commercial VR content. Unity has well-developed systems in place for rich graphics, realistic physics simulation, particles, animations, and more. Nevertheless, it does not contain any features specifically designed for the needs of human behavior researchers. We set out to produce an open-source software resource that would empower researchers to exploit the power of Unity for behavioral studies.

A literature search of human behavioral experiments reveals that experiments are often defined by a common model, one that more easily allows researchers to exercise the scientific method. Experiments are often composed of *trials*, which can be defined as an instance of a scenario. Trials are usually composed of a stimulus and a human response and are a basic unit of behavioral experiments. They can be repeated many times for a single participant, increasing the signal-to-noise ratio of measurements or allowing for the study of human behavior over time (e.g., adaptation and learning). *Blocks* can be defined as a grouping of trials that share something in common; comparing measures between blocks allows for the examination of how substantial changes to the scenario affect the response. A *session* is a single iteration of the task with a participant. Defining an experiment in such a session–block–trial model (Fig. 1) allows for the definition and communication of an experimental design without ambiguity.

**Fig. 1:**
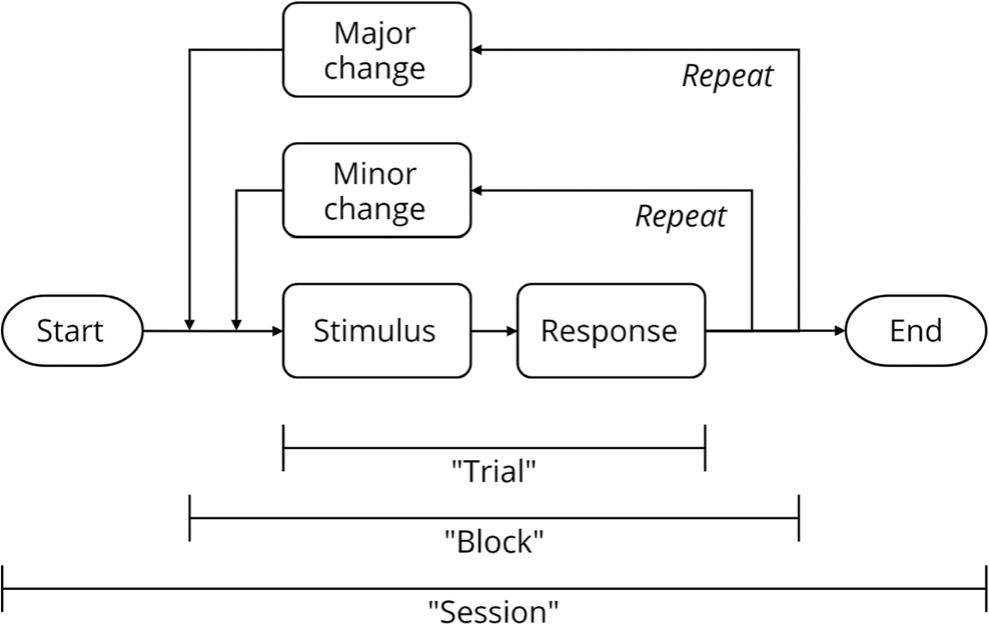
Structure of typical human behavior experiments, in the session–block–trial model. Many experiments comprise multiple repetitions of *trials*. Between trials, only minor changes are made. A substantial change of content in the trial is often described as creating a new *block*. A single iteration of a task by a participant is called a *session*.

The use of this session–block–trial model in computer-based experiments affords a certain type of system design structure that mirrors the model itself. Typically, the code produced for an experimental task consists of a loop, in which the process of presenting a stimulus and measuring a response is repeated many times, sometimes changing the parameters between loop iterations. The popularity of this experimental architecture means that researchers have attempted to provide tools that allow for the development of tasks without the need to “reinvent the wheel.” Relatedly, development of the stimuli for software experiments is often difficult without knowledge of low-level computer processes and hardware. Thus, several software packages have been released that aim to make the stimuli themselves easier to specify in code. There is some crossover between these two types of packages; some packages focus only on stimuli, whereas others also provide high-level ways to define the trials and blocks of the experiment. We briefly consider some of the most commonly used tools next.

PsychToolbox (Brainard, 1997) is a software package for MATLAB that allows researchers to program stimuli for vision experiments, providing the capability to perform low-level graphics operations but retaining the simplicity of the high-level interpreted MATLAB language. PsychoPy (Peirce, 2007) is an experimental control system that provides a means of using the Python programming language to systematically display stimuli to a user with precise timing. It consists of a set of common stimulus types, built-in functions for the collection and storage of user responses/behavior, and means of implementing various experimental design techniques (such as parameter staircases). PsychoPy also attempts to make research accessible for nonprogrammers with its “builder,” a graphical user interface (GUI) that allows the development of experiments with few to no computer programming requirements.

The graphics processes for immersive technologies are significantly more complex than those required for two-dimensional displays. In VR, it is difficult to think of stimuli in terms of a series of colored pixels. The additional complexity includes a need for stimuli to be displayed in apparent 3-D in order to simulate the naturalistic way objects appear to scale, move, and warp according to head position. Unity and other game engines have the capacity to implement the complex render pipeline that can accurately display stimuli in a virtual environment; however, current academic-focused visual display projects may not have the resources to keep up with the evolving demands of immersive technology software. Vizard (WorldViz, 2018), Unreal Engine (Epic Games, 2018), and open-source 3-D game engines such as Godot (Godot, 2018) and Xenko (Xenko, 2018) are also feasible alternatives to Unity, but Unity may still be a primary choice for researchers, because of its ease of use, maturity, and widespread popularity.

## The Unity Experiment Framework

To provide behavioral researchers with the power of Unity and the convenience of programs such as PsychoPy, we created the Unity Experiment Framework (UXF). UXF is a software framework for the development of human behavior experiments with Unity and the main programming language it uses, C#. UXF takes common programming concepts and features that are widely used and are often reimplemented for each experiment, and implements them in a generic fashion (Table 1). This gives researchers the tools to create their experimental software without the need to redevelop this common set of features. UXF aims specifically to solve this problem, and it deliberately excludes any kind of stimulus presentation system, with the view that Unity (and its large asset-developing community) can provide all the necessary means to implement any kind of stimulus or interaction system for an experiment. In summary, UXF provides the “nuts and bolts” that work behind the scenes of an experiment developed within Unity.

**Table 1:** Common experiment concepts and features that are represented in UXF

Concept	Description
Trial	The base unit of experiments. A trial is usually a singular attempt at a task by a participant after/during the presentation of a stimulus.
Block	A set of trials—often used to group consecutive trials that share something in common.
Session	A session encapsulates a full “run” of the experiment. Sessions are usually separated by a significant amount of time and could be within subjects (for the collection of data from a singular participant over several sessions) and/or between subjects (for the collection of data from several participants each carrying out a single session).
Settings	Settings are the parameters or variables for an experiment, block, or trial, usually predetermined, that quantitatively define the experiment. Settings are useful for defining the experimental manipulation (i.e., the independent variables).
Behavioral data	We perform an experiment to measure the effect of an independent variable on a dependent variable. Behavioral data collection allows for the collection of measured values of dependent variables on a trial-by-trial basis. For example, we may wish to collect the response to a multiple-choice question or the distance that a user throws a virtual ball.
Continuous data	Within a trial, we may want to measure a value of one or more parameters over time. Most commonly, we want to record the position and rotation of an object within each trial. This could be an object that is mapped to a real-world object (e.g., participant head, hands) or a fully virtual object (virtual ball in a throwing experiment). Tracking the position and rotation of an object is the main use case, but UXF supports the measurement of any parameter over time (e.g., pressure applied to a pressure pad).
Participant information	There may be other variables that we cannot control within the software, which we may wish to measure in order to record to examine a variable’s relationship to the result—for example, the age or gender of the participant.

### Experiment structure

UXF provides a set of high-level objects that directly map onto how we describe experiments. The goal is to make the experiment code more readable and avoid the temptation for inelegant if–else statements in the code as complexity increases. Sessions, blocks, and trials are the “objects” that can be represented within our code. The creation of a session, block, or trial automatically generates the properties we would expect them to have—for example, each block has a block number, and each trial has a trial number. These numbers are automatically generated as positive integers based on the order in which objects were created. Trials contain functionality such as “begin” and “end,” which will perform useful tasks implicitly in the background, such as recording the timestamp when the trial began or ended. Trials and blocks can be created programmatically, meaning that UXF can support any type of experiment structure, including staircase or adaptive procedures.

### Measuring dependent variables

While the trial is ongoing, at any point researchers can add any observations to the results of the trial, which will be added to the behavioral .CSV output data file at the end of the session. Additionally, a variable can be continuously logged over time at the same rate as the display refresh frequency (90 Hz in most currently available commercial VR HMDs). The main use case will be the position and rotation of any object in Unity, which can be automatically recorded on a per-trial basis, saving a single .CSV file for each trial of the session. This allows for easy cross-referencing with behavioral data. All data files (behavioral and continuous) are stored in a directory structure organized by *experiment > participant > session number*.

### Setting independent variables

**Fig. 2:**
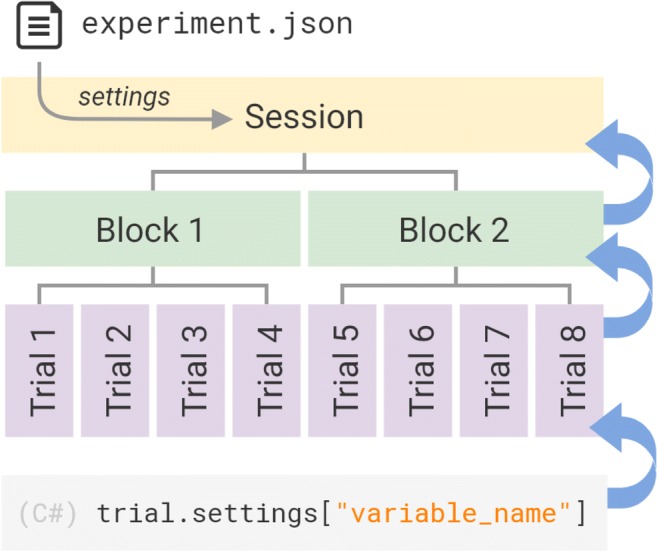
The UXF settings system. Independent variables that are changed as a means to iterate the design of an experiment, or to specify the experimental manipulation itself, can be written in a human-readable .json file. Settings can also be programmatically accessed or created at the trial, block, or session level. When a setting has not been specified, the request cascades up, so that the next level above is searched. This allows for both “gross” (e.g., to a whole session) and “fine” (e.g., to a single trial) storage of parameters within the same system.

Settings can be used to attach the values of an independent variable to an experiment, session, block, or trial. Settings have a cascading effect, whereby one can apply a setting to the whole session, a block, or a single trial. When attempting to access a setting, if the setting has not been assigned in the trial, UXF will attempt to access the setting in the block. If the setting has not been assigned in the block, UXF will search in the session (Fig. 2). This allows users to very easily implement features common to experiments, such as “10% of trials contain a different stimulus.” In this case, one could assign a “stimulus” setting for the whole session, but then assign 10% of the trials to have a different value for a “stimulus” setting.

Settings are also a useful feature to allow for changing experimental parameters without modifying the source code. A simple text file (.JSON format) can be placed in the experiment directory that will be read at the start of a session, and its settings will be applied to that session. This system speeds up the iteration time during the process of designing the experiment; the experimenter can change settings from this file and see their immediate effect, without changing any of the code itself. The system also allows multiple versions of the same experiment (e.g., different experimental manipulations) to be maintained within a single codebase using multiple settings files. One of these settings profiles can be selected by the experimenter on launching the experiment task.

### Experimenter user interface

**Fig. 3:**
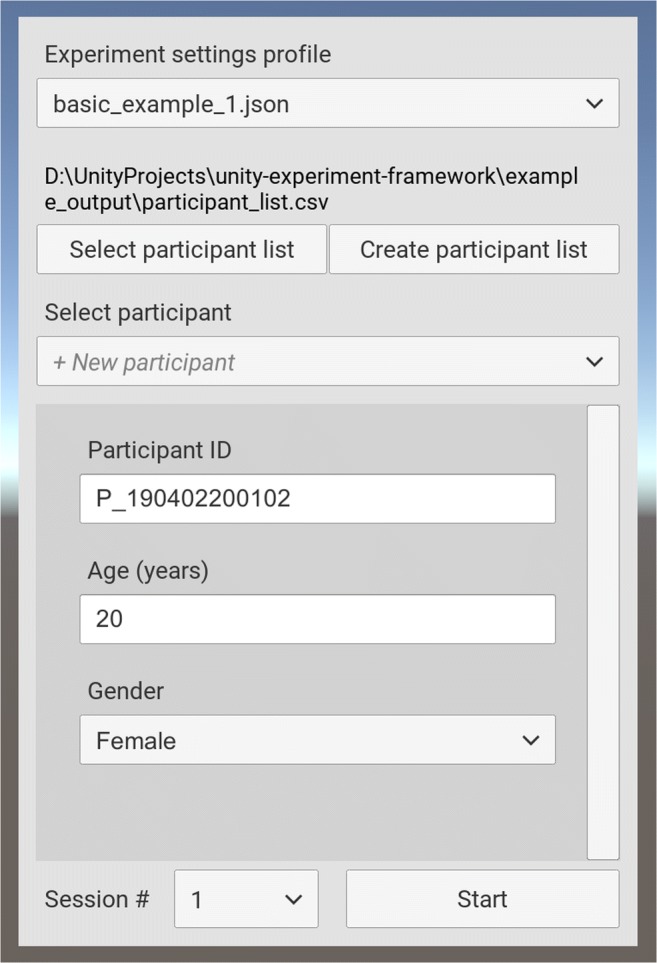
Screenshot of the experimenter user interface.

UXF includes an (optional) experimenter user interface (UI; Fig. 3) to allow selection of a settings profile and inputting of additional participant information, such as demographics. The information the experimenter wishes to collect is fully customizable. The UI includes support for a “participant list” system, whereby participant demographic information is stored in its own .CSV file. As new participants perform the experiment, their demographic information is stored in the list. This allows participant information to be more easily shared between sessions, or even separate experiments—instead of having to input the information each time, the experimenter can easily select any existing participant found in the participant list via a drop-down menu.

### Example

Below is an example of the C# code used to generate a simple two-block, ten-trial experiment in which the participant is presented with a number *x* and must input the doubled value (2*x*).

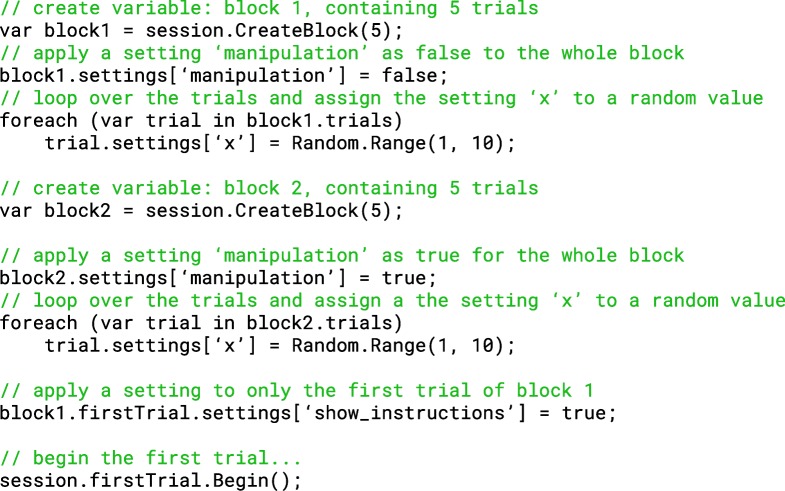



**Fig. 4:**
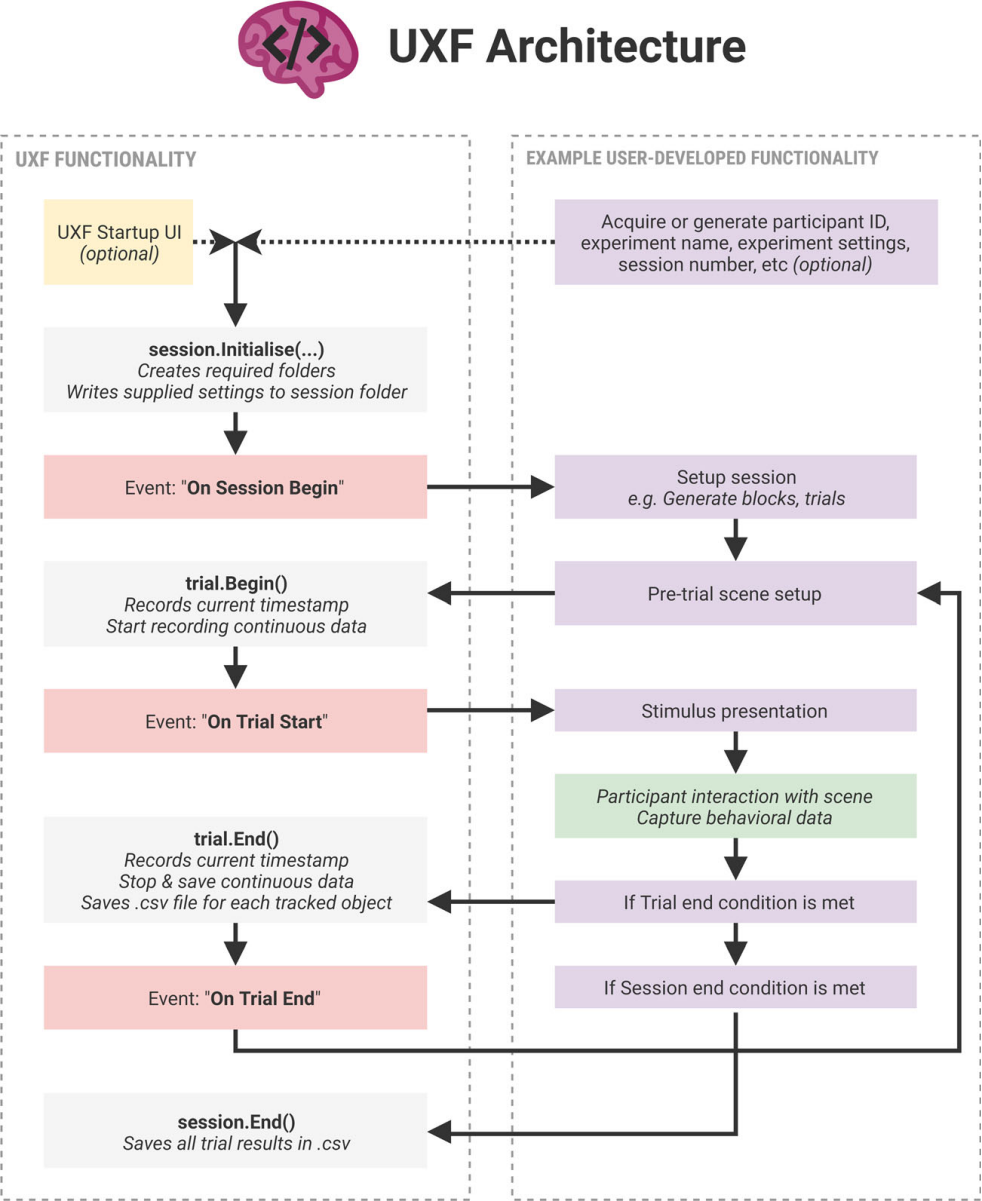
Structure of a typical task developed with UXF. The left panel shows the functionality present in UXF, with the functionality a researcher is expected to implement shown in the right panel. The framework features several “events” (shown in red), which are invoked at different stages during the experiment; these allow developers to easily add behaviors that occur at specific times—for example, presenting a stimulus at the start of a trial.

Elsewhere in our project, we must define what happens when we begin the trial (such as making the value of *x*x appear for the participant) and mechanisms to retrieve the participant’s response for the trial (i.e., the participant’s calculated value of 2*x*). These mechanisms are to be created with standard Unity features for making objects appear in the scene, collecting user response via keyboard input, and so forth. The resulting .CSV behavioral data file would be automatically generated and saved (Table 2). A typical structure of a task developed with UXF is shown in Fig. 4.

**Table 2:** Example behavioral data output

trial_num	block_num	start_time	end_time	manipulation	*x*	response
1	1	0.000	1.153	FALSE	8	16
2	1	1.153	2.112	FALSE	3	6
3	1	2.112	2.950	FALSE	4	8
4	1	2.950	3.921	FALSE	7	14
5	1	3.921	4.727	FALSE	4	8
6	2	4.727	5.826	TRUE	9	18
7	2	5.826	6.863	TRUE	5	10
8	2	6.863	7.693	TRUE	10	20
9	2	7.693	8.839	TRUE	6	12
10	2	8.839	9.992	TRUE	3	6

Columns not shown include participant ID, session number, and experiment name.

### Multithreading file input/output (I/O)

Continuous measurement of variables requires that large amounts of data be collected over the course of the experiment. When using a VR HMD, it is essential to maintain a high frame rate and keep stutters to a minimum in order to minimize the risk of inducing sickness or discomfort in the participant. Handling of tasks such as reading and writing to a file may take several milliseconds or more, depending on the operating system’s background work. Constant data collection (particularly when tracking the movement of many objects in the scene) and writing these data to file therefore poses a risk of dropping the frame rate below acceptable levels. The solution is to create a multithreaded application that allows the virtual environment to continue to be updated while data are being written to files simultaneously in a separate thread. Designing a stable multithreaded application imparts additional technical requirements on the researcher. UXF abstracts file I/O away from the developer, performing these tasks automatically, with a multithreaded architecture working behind the scenes. Additionally, the architecture contains a queuing system, where UXF queues up all data tasks and writes the files one by one, even halting the closing of the program in order to finish emptying the queue, if necessary.

### Cloud-based experiments

**Fig. 5:**
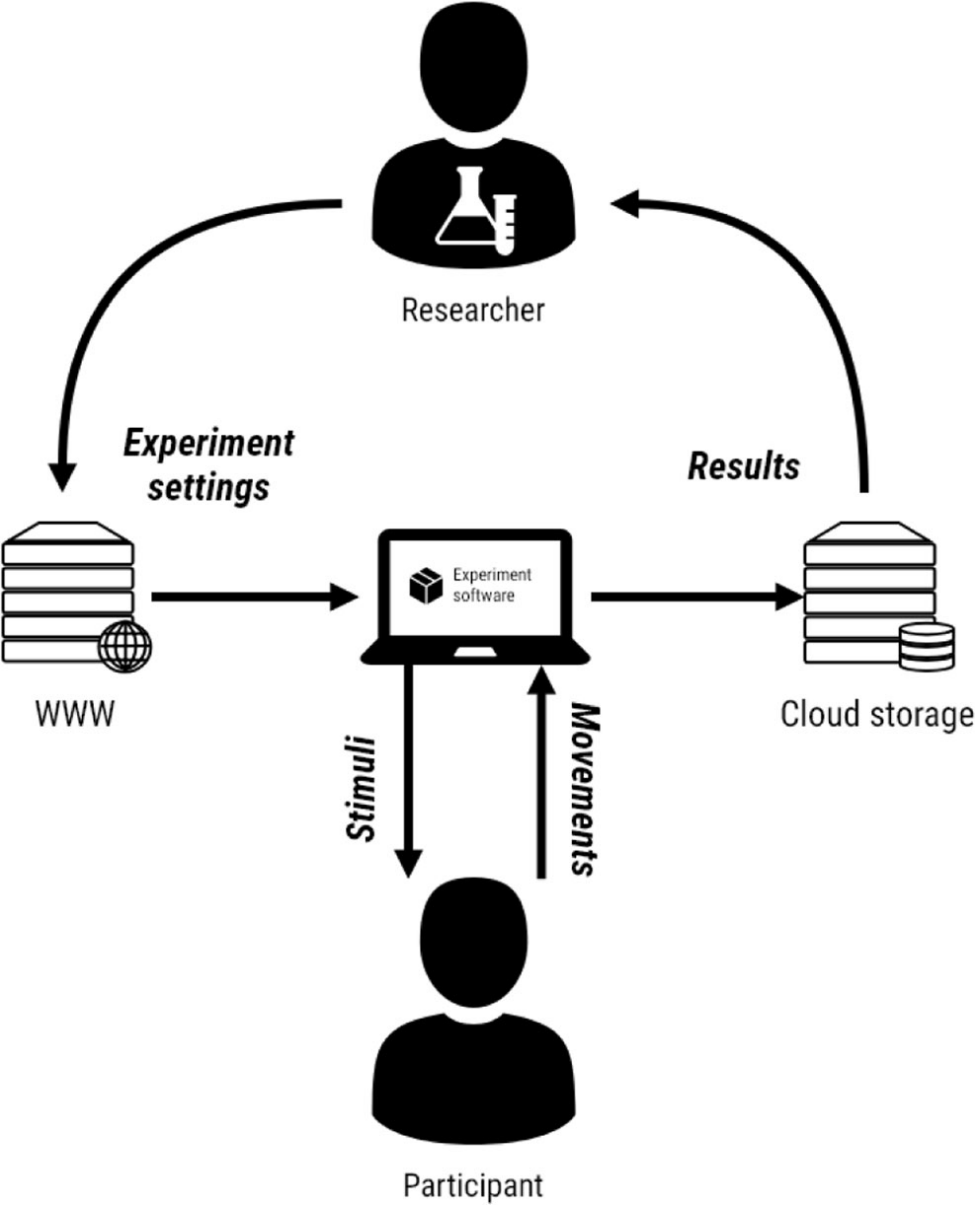
Experiment in the cloud. A piece of software developed with UXF can be deployed to an internet-connected device. Researchers can modify the experiment settings to test different experimental manipulations over time, which are downloaded from the web by the client device upon running a UXF experiment. As the participant partakes in the experiment, stimuli are presented, and the participant’s movements are recorded in the form of behaviors/responses or continuous measurement of such parameters as hand position. The results are automatically and securely streamed to a server on the internet, from which the researcher can periodically retrieve the data.

UXF is a standalone, generic project, so it does not put any large design constraints on developers using it. This means that UXF does not have to be used in a traditional lab-based setting, with researchers interacting directly with participants; it can also be used for data collection opportunities outside the lab, by embedding experiments within games or apps that a user can partake in at their discretion. Data are then sent to a web server, from which they can later be downloaded and analyzed by researchers (Fig. 5). Recently these cloud-based experiments have become a viable method of performing experiments on a large scale.

UXF can be used in cloud-based experiments (Fig. 5) using two independent pieces of software that accompany UXF:

1. *UXF S3 Uploader* allows all files that are saved by UXF (behavioral data, continuous data, and logs) to be additionally uploaded to a location in Amazon’s Simple Storage Service, as set up by a researcher. This utilizes the existing UXF functionality of setting up actions to be executed after a file has been written, so a developer could potentially implement uploading the files to any other storage service.
2. *UXF Web Settings* replaces the default UXF functionality of selecting experiment settings via a user interface, allowing settings instead to be accessed automatically from a web URL by the software itself. This allows a deployed experiment (e.g., via an app store, or simply by transferring an executable file) to be remotely altered by the researcher, without any modification to the source code. Settings files are stored in .json format and would usually be of very small file size, so they can be hosted online cheaply and easily.

A developer can implement neither, either, or both of these extras, depending on the needs of the research. For lab-based experiments, neither is required. For experiments without any need to modify settings afterward, but with the requirement of securely backing up data in the cloud, the first option can be used. If a researcher wants to remotely modify settings but has physical access to the devices to retrieve data, the second option can be used. For a fully cloud-based experiment without direct researcher contact with the participant, both optional functionalities can be used. This has been successfully tried and tested, in the context of a museum exhibition in which visitors could take part in VR experiments, with the recorded data being uploaded to the internet. Both UXF S3 Uploader and UXF Web Settings are available as open-source Unity packages.

## Case study

One classic question in human behavioral research is related to the information used by adults and children when maintaining posture (Edwards, 1946; Thomas & Whitney, 1959). To investigate the contributions of kinesthetic and vision information when both are available, four decades ago Lee and Aronson (1974) used a physical “swinging” room to perturb the visual information provided by the walls and ceiling while leaving the kinesthetic information unaffected (only the walls and ceiling swung; the floor did not move). This experiment demonstrated the influence of vision on posture, but the scale of the apparatus meant that it could only ever be implemented in a laboratory setting. The approach was also subject to both measurement errors and researcher bias (Wann, Mon-Williams, & Rushton 1998). More recently, conventional computer displays have been used to explore the impact of vision on posture (e.g., Villard, Flanagan, Albanese, & Stoffregen, 2008), and this method has addressed issues of measurement error and researcher bias, but still it remains confined to the laboratory.

The ability to create a virtual swinging room in a VR environment provides a test case for the use of UXF to support behavioral research and provides a proof-of-concept demonstration of how large laboratory experiments can be placed within a nonlaboratory setting. Here, we used the head-tracking function as a proxy measure of postural stability (since decreased stability would be associated with more head sway; Flatters et al., 2014). To test the UXF software, we constructed a simple experiment with both a within-participant component (whether the virtual room was stationary or oscillating) and a between-participant factor (adults vs. children). We then deployed the experiment in a museum with a trained demonstrator and remotely collected data from 100 participants.

The task was developed in the Unity game engine, with UXF handling several aspects of the experiment, including participant information collection, settings, behavioral data, and continuous data.

- *Participant information collection*: The UXF built-in user interface was used to collect a unique participant ID as well as the participant’s age and gender. This information was stored in a .CSV participant list file. This list was subsequently updated with participant height and arm span as they were collected in the task.
- *Settings*: A settings file accompanied the task, which allowed modification of the assessment duration as well as the oscillation amplitude and period without modifying the code. The settings for each trial were used to construct the environment in order to facilitate the requested trial condition.
- *Behavioral data*: Although no dependent variables were directly measured on each trial, the UXF behavioral data collection system output a list of all trials that were run in that session, as well as the vision condition for each trial.
- *Continuous data*: UXF was configured to automatically log the HMD position over time within each trial, which was then used offline for the stability measure calculation. UXF split the files, with one file per trial, which was designed to make it easy to match each file with the trial condition under which the file was collected.

### Method

Fifty children (all under 16 years of age; mean age = 9.6 years, *SD* = 2.0 years) and 50 adults (mean age = 27.5 years, *SD* = 13.2 years) took part in the study. The participants either were recruited from the University of Leeds participant pool (adults) or were attendees at the Eureka! Science Museum (children and adults) and provided full consent. A gaming-grade laptop (Intel Core i5-7300HQ, Nvidia GTX 1060), a VR HMD (Oculus Rift CV1), and the SteamVR application program interface (API), a freely available package independent of UXF (Valve Corp., 2018), were used to present the stimuli and collect data. The HMD was first calibrated using the built-in procedure, which set the virtual floor level to match the physical floor.

**Fig. 6:**
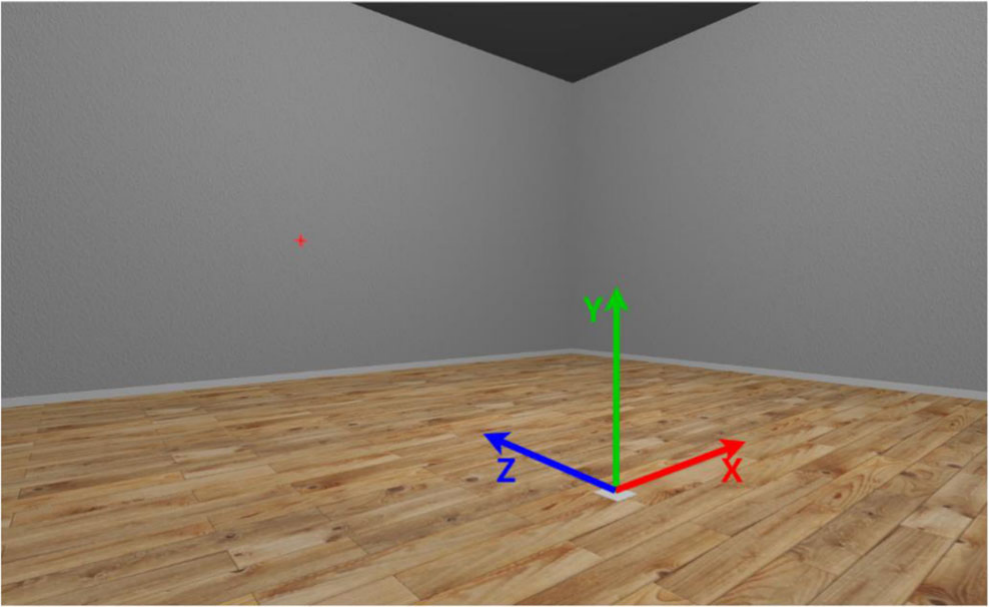
Screenshot from inside the virtual room. Arrows indicate the three axes as well as the origin. The red fixation cross is shown on the wall.

After explaining the task requirements, the demonstrator put the HMD on the participant’s head (over glasses, if necessary) and adjusted it until the participant reported that it was comfortable and they could see clearly. Participants were then placed in the center of a simple virtual room (height 3 m, width 6 m, depth 6 m) with textured walls and floors (Fig. 6). Height was measured as vertical distance from the floor to the “center eye” of the participant (as reported by the SteamVR API), and this value was used to place a fixation cross on the wall at the participant’s height.

The task comprised two 10-s trials performed in a random order. The *normal* condition asked participants to stand still and look at a fixation cross placed on the wall. In the *oscillating* condition, participants were given the same instructions, but the virtual room oscillated in a sinusoidal fashion (rotating around the *x*-axis) with an amplitude of 5° and a frequency of 0.25 Hz. The oscillation was performed about the point on the floor at the center of the room, in effect keeping the participant’s feet fixed in place. Participants were not explicitly informed about the room oscillation. The position of the HMD inside the virtual room was logged at a rate of 90 Hz during each of the two trials. The path length of the head was used as a proxy measure of postural stability (sum of all point-to-point distances over a trial).

### Results

**Fig. 7:**
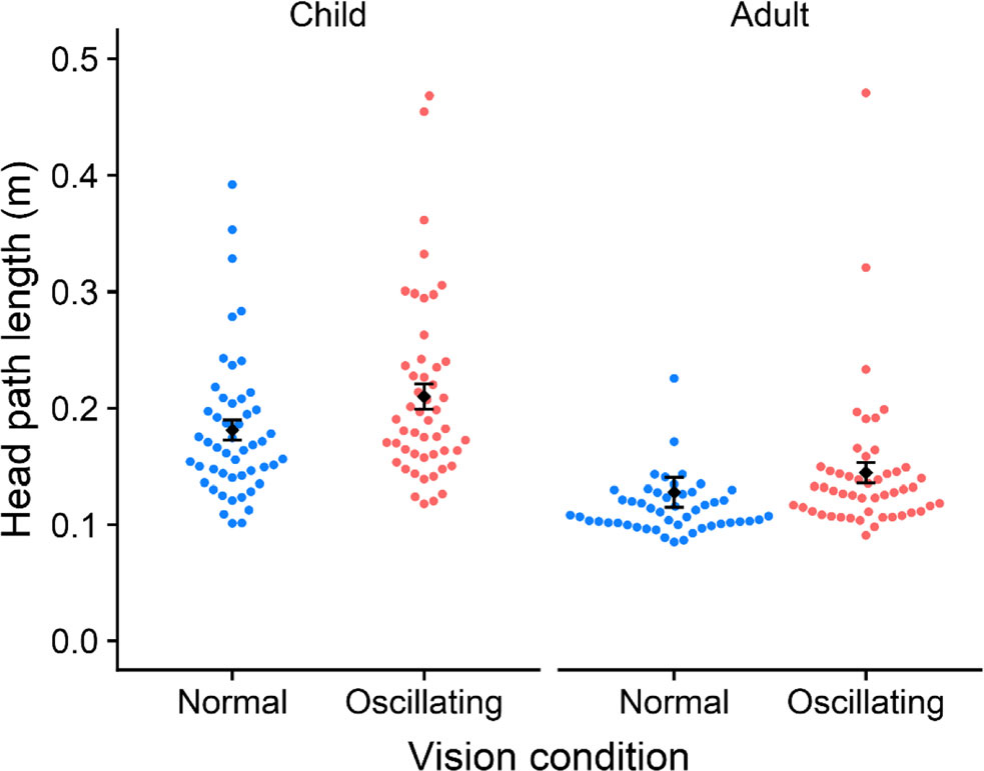
Head path length (where higher values indicate worse postural stability) as a function of vision condition. The two conditions were “normal” (static virtual room) and “oscillating” (oscillating virtual room). Postural stability was indexed by the path length of head movement, in meters (measured over a 10-s period). Adults showed a significantly different path length overall, as compared to children (shorter, indicating greater stability). Error bars represent ± 1 *SEM*.

No participants reported any feelings of sickness or discomfort during or after taking part in the task. A mixed-model design analysis of variance (ANOVA; 2 [Age: adult vs. children] × 2 Vision Condition [normal vs. oscillating]) revealed no interaction, *F*(2, 98) = 0.34, *p* = .562, *η*^2^_G_ = .001, but it did reveal main effects of vision, *F*(2, 98) = 7.35, *p* = .008, *η*^2^_G_ = .016, and age, *F*(1, 98) = 9.26, *p* = .003, *η*^2^_G_ = .068, thus replicating previous work on the contribution of visual information to postural stability (Flatters et al., 2014) (see Fig. 7).

## Summary

We have created an open-source resource that enables researchers to use the powerful gaming engine Unity when designing experiments. We tested the usefulness of UXF by designing an experiment that could be deployed within a museum setting. We found that UXF simplified the development of the experiment and produced measures, in the form of data files that were in a format that made subsequent data analysis straightforward. The data collected were consistent with the equivalent laboratory-based measures (reported over many decades of research), whereby children showed less postural stability than did adults, and whereby both adults and children showed greater sway when the visual information was perturbed. There are likely to be differences in the postural responses of both adults and children within a virtual environment relative to a laboratory setting, and we do not suggest that the data are quantitatively similar between these settings. Nonetheless, these data do show that remotely deployed VR systems can capture age differences and detect the outcomes of an experimental manipulation.

Our planned work includes maintaining the software for compatibility with future versions of Unity and refactoring UXF so that it works on a wider range of platforms (e.g., mobile devices, web browsers, augmented-reality devices, and standalone VR headsets). Features may be added or modified if a clear need arises. The project is open-source, thus allowing researchers in the field to implement and share such additions.

### Availability

UXF is freely available to download via GitHub as a Unity package (github.com/immersivecognition/unity-experiment-framework) and currently can be integrated into Unity tasks built for Windows PCs. Documentation and support is available on the GitHub wiki (github.com/immersivecognition/unity-experiment-framework/wiki). The package is open-source under the MIT license. The related packages UXF S3 Uploader and UXF Web Settings are available via the same GitHub link.

### Author note

The authors thank Almanzo McConkey and Andrea Loriedo for their feedback on earlier versions of the software. Authors F.M. and M.M.-W. hold Fellowships from the Alan Turing Institute. M.W., F.M., and M.M.-W. are supported by a Research Grant from the EPSRC (Grant EP/R031193/1).
